# Supplementary material for: TGF-β reduces DNA ds-break repair mechanisms to heighten genetic diversity and adaptability of CD44+/CD24− cancer cells
Source: eLife. 2017 Jan 16;6:e21615. doi: 10.7554/eLife.21615 (PMC5345931; doi:10.7554/eLife.21615)
Supplement: Supplementary file 1. — COSMIC (Catalogue Of Somatic Mutations In Cancer). http://cancer.sanger.ac.uk/cosmic DOI: http://dx.doi.org/10.7554/eLife.21615.031 [file elife-21615-supp1.pdf]

Supplementary File 1

Mutation analysis of the following cells lines (used in our study) from COSMIC (Catalogue Of Somatic Mutations In Cancer http://cancer.sanger.ac.uk/cosmic

Cell lines » Sample » Overview » NCI-H1650

View in GRCh37 Ar

Circos | Genome Browser | Overview | Mutations | Fusions | Mutation Spectrum | Sequence Context | Heatmap | Breakpoints | Non-Coding mutation | CNV & Expr | Methylation

Show 10 entries Search: Export: CSV TSV

Gene	Transcript	Census Gene	AA Mutation	CDS Mutation	Somatic status	Zygosity	Validated	Type
APC	ENST00000457016	Yes	p.A1358T	c.4072G>A	Unknown	Heterozygous	Unverified	Substitution - Missense
CAMTA1	ENST00000303635	Yes	p.S239L	c.716C>T	Unknown	Heterozygous	Unverified	Substitution - Missense
DEK	ENST00000397239	Yes	p.K221K	c.663A>G	Unknown	Homozygous	Unverified	Substitution - coding silent
EGFR	ENST00000275493	Yes	p.E746_A750delELREA	c.2235_2249del15	Confirmed Somatic	Heterozygous	Verified	Deletion - In frame
NF1	ENST00000358273	Yes	p.?	c.3114-7T>C	Unknown	Heterozygous	Unverified	Unknown
SBDS	ENST00000246868	Yes	p.I133V	c.397A>G	Unknown	Heterozygous	Unverified	Substitution - Missense
SFPQ	ENST00000357214	Yes	p.A651A	c.1953A>T	Unknown	Heterozygous	Unverified	Substitution - coding silent
TP53	ENST00000269305	Yes	p.?	c.673-2A>G	Confirmed Somatic	Homozygous	Verified	Unknown

Showing 1 to 8 of 8 entries First Previous 1 Next Last

Cell lines » Sample » Overview » A549

View in GR

Circos | Genome Browser | Overview | Mutations | Fusions | Mutation Spectrum | Sequence Context | Heatmap | Breakpoints
Non-Coding mutation | CNV & Expr | Methylation

Show 25 entries Search: Export:

Gene	Transcript	Census Gene	AA Mutation	CDS Mutation	Somatic status	Zygosity	Validated	Type
ATR	NM_001184	Yes	p.?	c.2634-1G>A	Unknown	Heterozygous	Unverified	Unknown
ATR	NM_001184	Yes	p.Q1441Q	c.4323A>G	Unknown	Heterozygous	Unverified	Substitution - coding silent
CARD11	ENST00000396946	Yes	p.M1124I	c.3372G>A	Unknown	Heterozygous	Unverified	Substitution - Missense
CBL	ENST00000264033	Yes	p.R585C	c.1753C>T	Unknown	Homozygous	Unverified	Substitution - Missense
CTNNA1	ENST00000302763	Yes	p.E865*	c.2593G>T	Unknown	Heterozygous	Unverified	Substitution - Nonsense
ERC1	ENST00000397203	Yes	p.T1032T	c.3096C>A	Unknown	Homozygous	Unverified	Substitution - coding silent
FH	ENST00000366560	Yes	p.G464V	c.1391G>T	Previously Reported	Heterozygous	Unverified	Substitution - Missense
FLT3	ENST00000241453	Yes	p.N323K	c.969C>A	Unknown	Heterozygous	Unverified	Substitution - Missense
FUS	ENST00000254108	Yes	p.R383H	c.1148G>A	Unknown	Heterozygous	Unverified	Substitution - Missense
HIP1	ENST00000336926	Yes	p.S277N	c.830G>A	Previously Reported	Heterozygous	Unverified	Substitution - Missense
KRAS	ENST00000311936	Yes	p.G12S	c.34G>A	Confirmed Somatic	Homozygous	Verified	Substitution - Missense
PCSK7	ENST00000320934	Yes	p.?	c.1156-9C>T	Unknown	Homozygous	Unverified	Unknown
POLE	ENST00000320574	Yes	p.Q1475Q	c.4425G>A	Unknown	Heterozygous	Unverified	Substitution - coding silent
SMARCA4	ENST00000429416	Yes	p.Q729fs*4	c.2184_2206del23	Confirmed Somatic	Heterozygous	Verified	Deletion - Frameshift
SMARCA4	ENST00000429416	Yes	p.Q729fs*4	c.2184_2206del23	Previously Reported	Homozygous	Verified	Deletion - Frameshift
STK11	ENST00000326873	Yes	p.Q37*	c.109C>T	Confirmed Somatic	Homozygous	Verified	Substitution - Nonsense
SUFU	ENST00000369902	Yes	p.T411M	c.1232C>T	Previously Reported	Heterozygous	Unverified	Substitution - Missense
TBL1XR1	ENST00000430069	Yes	p.T290A	c.868A>G	Unknown	Homozygous	Unverified	Substitution - Missense
USP6	NM_004505.1	Yes	p.P31T	c.91C>A	Unknown	Heterozygous	Unverified	Substitution - Missense

Showing 1 to 19 of 19 entries

First Previous 1 Next

Supplementary File 1

Cell lines » Sample » Overview » MCF7

View in GRCh37 Archive

Circos | Genome Browser | Overview | **Mutations** | Fusions | Mutation Spectrum | Sequence Context | Heatmap | Breakpoints | Non-Coding mutation | CNV & Expr | Methylation

Show 25 entries Search: Export: CSV TSV

Gene	Transcript	Census Gene	AA Mutation	CDS Mutation	Somatic status	Zygosity	Validated	Type
ATP2B3	ENST00000359149	Yes	p.V882E	c.2645T>A	Previously Reported	Heterozygous	Unverified	Substitution - Missense
BCR	ENST00000305877	Yes	p.I456T	c.1367T>C	Unknown	Heterozygous	Unverified	Substitution - Missense
CLIP1	ENST00000358808	Yes	p.N890S	c.2669A>G	Unknown	Heterozygous	Unverified	Substitution - Missense
DNM2	ENST00000355667	Yes	p.A800V	c.2399C>T	Previously Reported	Heterozygous	Unverified	Substitution - Missense
ECT2L	ENST00000367682	Yes	p.T727T	c.2181C>T	Unknown	Heterozygous	Unverified	Substitution - coding silent
EP300	ENST00000263253	Yes	p.R1356*	c.4066C>T	Previously Reported	Heterozygous	Unverified	Substitution - Nonsense
FLT3	ENST00000241453	Yes	p.C368Y	c.1103G>A	Unknown	Heterozygous	Unverified	Substitution - Missense
FLT3	ENST00000241453	Yes	p.C368S	c.1102T>A	Unknown	Heterozygous	Unverified	Substitution - Missense
GATA3	ENST00000379328	Yes	p.D336fs*17	c.1006_1007insG	Previously Reported	Heterozygous	Unverified	Insertion - Frameshift
MAP3K13	ENST00000424227	Yes	p.D380N	c.1138G>A	Previously Reported	Heterozygous	Unverified	Substitution - Missense
MYH9	ENST00000216181	Yes	p.K682_L687delKLDPHL	c.2044_2061del18	Unknown	Heterozygous	Unverified	Deletion - In frame
NBN	ENST00000265433	Yes	p.R43*	c.127C>T	Unknown	Heterozygous	Unverified	Substitution - Nonsense
PALB2	ENST00000261584	Yes	p.V560L	c.1678G>C	Unknown	Heterozygous	Unverified	Substitution - Missense
PIK3CA	NM_006218.1	Yes	p.E545K	c.1633G>A	Confirmed Somatic	Heterozygous	Verified	Substitution - Missense

Showing 1 to 14 of 14 entries First Previous 1 Next Last

Supplementary File 1

Cell lines » Sample » Overview » BT-474

View in GRCh37 Archi

Circos | Genome Browser | Overview | **Mutations** | Fusions | Mutation Spectrum | Sequence Context | Heatmap | Breakpoints | Non-Coding mutation | CNV & Expr | Methylation

Show 100 entries   Search: Export: CSV TSV

Gene	Transcript	Census Gene	AA Mutation	CDS Mutation	Somatic status	Zygosity	Validated	Type
AFF3	ENST00000409579	Yes	p.N983N	c.2949C>T	Unknown	Heterozygous	Unverified	Substitution - coding silent
AFF3	ENST00000409579	Yes	p.S1112*	c.3335C>G	Unknown	Heterozygous	Unverified	Substitution - Nonsense
ARHGEF12	ENST00000397843	Yes	p.Q933E	c.2797C>G	Unknown	Heterozygous	Unverified	Substitution - Missense
ATM	ENST00000278616	Yes	p.E2468K	c.7402G>A	Unknown	Heterozygous	Unverified	Substitution - Missense
AXIN1	ENST00000262320	Yes	p.?	c.2186+8C>T	Unknown	Heterozygous	Unverified	Unknown
BCOR	ENST00000397354	Yes	p.H457P	c.1370A>C	Unknown	Homozygous	Unverified	Substitution - Missense
BRCA2	ENST00000380152	Yes	p.S3094*	c.9281C>A	Unknown	Heterozygous	Unverified	Substitution - Nonsense
BRIP1	ENST00000259008	Yes	p.S230L	c.689C>T	Unknown	Heterozygous	Unverified	Substitution - Missense
BUB1B	ENST00000287598	Yes	p.S521F	c.1562C>T	Unknown	Heterozygous	Unverified	Substitution - Missense
CARD11	ENST00000396946	Yes	p.L15L	c.45G>A	Previously Reported	Heterozygous	Unverified	Substitution - coding silent
CCND1	ENST00000227507	Yes	p.L217L	c.651G>C	Unknown	Heterozygous	Unverified	Substitution - coding silent
CIC	ENST00000160740	Yes	p.L1467V	c.4399C>G	Unknown	Heterozygous	Unverified	Substitution - Missense
COL2A1	ENST00000337299	Yes	p.G669S	c.2005G>A	Unknown	Heterozygous	Unverified	Substitution - Missense
EGFR	ENST00000275493	Yes	p.D1014D	c.3042C>T	Unknown	Heterozygous	Unverified	Substitution - coding silent
EPS15	ENST00000371733	Yes	p.G522E	c.1565G>A	Previously Reported	Heterozygous	Unverified	Substitution - Missense
FANCA	ENST00000389301	Yes	p.L684L	c.2052G>A	Unknown	Heterozygous	Unverified	Substitution - coding silent
FANCA	ENST00000389301	Yes	p.S1114fs*3	c.3340delT	Unknown	Heterozygous	Unverified	Deletion - Frameshift
FLT4	ENST00000393347	Yes	p.?	c.3431+7C>T	Unknown	Heterozygous	Unverified	Unknown
FLT4	ENST00000393347	Yes	p.A634T	c.1900G>A	Unknown	Heterozygous	Unverified	Substitution - Missense
GNA11	ENST00000078429	Yes	p.I167I	c.501C>T	Unknown	Homozygous	Unverified	Substitution - coding silent
HIST1H3B	ENST00000244661	Yes	p.R135T	c.404G>C	Unknown	Heterozygous	Unverified	Substitution - Missense
HNF1A	ENST00000257555	Yes	p.Q495*	c.1483C>T	Unknown	Heterozygous	Unverified	Substitution - Nonsense
HOXA13	ENST00000222753	Yes	p.S314L	c.941C>T	Unknown	Heterozygous	Unverified	Substitution - Missense
KIF5B	ENST00000302418	Yes	p.Q734E	c.2200C>G	Unknown	Heterozygous	Unverified	Substitution - Missense
KMT2D	ENST00000301067	Yes	p.E1350K	c.4048G>A	Unknown	Heterozygous	Unverified	Substitution - Missense
NCOR1	ENST00000268712	Yes	p.?	c.6734-3C>T	Unknown	Heterozygous	Unverified	Unknown
NSD1	ENST00000439151	Yes	p.S1654C	c.4961C>G	Unknown	Heterozygous	Unverified	Substitution - Missense
NSD1	ENST00000439151	Yes	p.L2063V	c.6187C>G	Unknown	Heterozygous	Unverified	Substitution - Missense
NUMA1	ENST00000393695	Yes	p.E1479Q	c.4435G>C	Unknown	Homozygous	Unverified	Substitution - Missense
PAX3	ENST00000392069	Yes	p.P373P	c.1119G>A	Unknown	Heterozygous	Unverified	Substitution - coding silent
PDE4DIP	ENST00000369354	Yes	p.D371N	c.1111G>A	Unknown	Heterozygous	Unverified	Substitution - Missense
PIK3CA	NM_006218.1	Yes	p.K111N	c.333G>C	Confirmed Somatic	Heterozygous	Verified	Substitution - Missense

Gene	Transcript	Census Gene	AA Mutation	CDS Mutation	Somatic status	Zygosity	Validated	Type
PLAG1	ENST00000316981	Yes	p.D14H	c.40G>C	Unknown	Heterozygous	Unverified	Substitution - Missense
PML	ENST00000268058	Yes	p.L869R	c.2606T>G	Unknown	Heterozygous	Unverified	Substitution - Missense
PMS1	ENST00000441310	Yes	p.K156R	c.467A>G	Unknown	Heterozygous	Unverified	Substitution - Missense
PPFIBP1	ENST00000228425	Yes	p.V122M	c.364G>A	Unknown	Heterozygous	Unverified	Substitution - Missense
PPP2R1A	ENST00000322088	Yes	p.S143F	c.428C>T	Previously Reported	Heterozygous	Unverified	Substitution - Missense
PTPRK	ENST00000368226	Yes	p.D884N	c.2650G>A	Unknown	Heterozygous	Unverified	Substitution - Missense
RAP1GDS1	ENST00000453712	Yes	p.H122Y	c.364C>T	Unknown	Heterozygous	Unverified	Substitution - Missense
RECQL4	ENST00000617875	Yes	p.M989R	c.2966T>G	Unknown	Heterozygous	Unverified	Substitution - Missense
RHOA	ENST00000418115	Yes	p.G17E	c.50G>A	Previously Reported	Heterozygous	Unverified	Substitution - Missense
RNF213	ENST00000336301	Yes	p.V1085V	c.3255C>T	Unknown	Heterozygous	Unverified	Substitution - coding silent
RNF213	ENST00000336301	Yes	p.L1176L	c.3528C>G	Unknown	Heterozygous	Unverified	Substitution - coding silent
ROS1	ENST00000368508	Yes	p.V1916L	c.5746G>T	Unknown	Heterozygous	Unverified	Substitution - Missense
SMARCB1	ENST00000263121	Yes	p.F328F	c.984C>T	Unknown	Heterozygous	Unverified	Substitution - coding silent
SS18	ENST00000415083	Yes	p.D293N	c.877G>A	Unknown	Heterozygous	Unverified	Substitution - Missense
SSX1	ENST00000376919	Yes	p.E149G	c.446A>G	Previously Reported	Heterozygous	Unverified	Substitution - Missense
STAT3	ENST00000264657	Yes	p.F174L	c.522C>G	Unknown	Heterozygous	Unverified	Substitution - Missense
TFRC	ENST00000360110	Yes	p.V108V	c.324G>T	Unknown	Heterozygous	Unverified	Substitution - coding silent
TFRC	ENST00000360110	Yes	p.?	c.1405-9C>G	Unknown	Heterozygous	Unverified	Unknown
TP53	ENST00000269305	Yes	p.E285K	c.853G>A	Confirmed Somatic	Homozygous	Verified	Substitution - Missense
TPM3	ENST00000368533	Yes	p.R208fs*10	c.623_624delGA	Unknown	Heterozygous	Unverified	Deletion - Frameshift
WRN	ENST00000298139	Yes	p.E764G	c.2291A>G	Unknown	Heterozygous	Unverified	Substitution - Missense
WRN	ENST00000298139	Yes	p.H945N	c.2833C>A	Unknown	Heterozygous	Unverified	Substitution - Missense

Cosmic » Sample » Overview » PC9

View in GRCh37

Overview | Mutations | Fusions | Non-Mutant Genes | CNV & Expr | Methylation | Studies | References

Show 10 entries   Search: Export: CSV

Gene	Transcript	Census Gene	AA Mutation	CDS Mutation	Somatic status	Zygosity	Validated	Type
EGFR	ENST00000275493	Yes	p.E746_A750delELREA	c.2235_2249del15	Previously Reported	Heterozygous	Unverified	Deletion - In frame

Showing 1 to 1 of 1 entries

First | Previous | 1 | Next | La

Supplementary File 1

Cell lines » Sample » Overview » NCI-H23

View in GRCh37 Archive

Circos | Genome Browser | Overview | Mutations | Fusions | Mutation Spectrum | Sequence Context | Heatmap | Breakpoints | Non-Coding mutation | CNV & Expr | Methylation

Show 50 entries Search: Export: CSV TSV

Gene	Transcript	Census Gene	AA Mutation	CDS Mutation	Somatic status	Zygosity	Validated	Type
ACSL6	ENST00000296869	Yes	p.?	c.1507+1G>T	Unknown	Heterozygous	Unverified	Unknown
ALK	ENST00000389048	Yes	p.L1035L	c.3105G>A	Unknown	Heterozygous	Unverified	Substitution - coding silent
ARID1B	ENST00000275248	Yes	p.A1917A	c.5751G>T	Unknown	Homozygous	Unverified	Substitution - coding silent
ARID2	ENST00000334344	Yes	p.M452V	c.1354A>G	Previously Reported	Heterozygous	Unverified	Substitution - Missense
ASXL1	ENST00000375687	Yes	p.Y1087H	c.3259T>C	Unknown	Heterozygous	Unverified	Substitution - Missense
ATM	ENST00000278616	Yes	p.Q1919P	c.5756A>C	Unknown	Homozygous	Unverified	Substitution - Missense
AXIN2	ENST00000307078	Yes	p.T586T	c.1758G>A	Unknown	Heterozygous	Unverified	Substitution - coding silent
BCR	ENST00000305877	Yes	p.R567H	c.1700G>A	Unknown	Heterozygous	Unverified	Substitution - Missense
BRIP1	ENST00000259008	Yes	p.E1054A	c.3161A>C	Unknown	Heterozygous	Unverified	Substitution - Missense
CCNE1	ENST00000262643	Yes	p.D83N	c.247G>A	Unknown	Heterozygous	Unverified	Substitution - Missense
CIC	ENST00000160740	Yes	p.S1031S	c.3093G>C	Unknown	Homozygous	Unverified	Substitution - coding silent
CSF1R	ENST00000286301	Yes	p.R710R	c.2130C>T	Unknown	Heterozygous	Unverified	Substitution - coding silent
CSF1R	ENST00000286301	Yes	p.P202S	c.604C>T	Unknown	Heterozygous	Unverified	Substitution - Missense
DDX6	NM_004397.3	Yes	p.?	c.1141+5G>A	Unknown	Heterozygous	Unverified	Unknown
DICER1	ENST00000343455	Yes	p.E732E	c.2196A>G	Unknown	Heterozygous	Unverified	Substitution - coding silent
DNMT3A	ENST00000321117	Yes	p.G223*	c.667G>T	Previously Reported	Heterozygous	Unverified	Substitution - Nonsense
EBF1	ENST00000313708	Yes	p.P339Q	c.1016C>A	Previously Reported	Heterozygous	Unverified	Substitution - Missense
FGFR2	ENST00000358487	Yes	p.Y328S	c.983A>C	Unknown	Homozygous	Unverified	Substitution - Missense
FLT3	ENST00000241453	Yes	p.P738H	c.2213C>A	Unknown	Heterozygous	Unverified	Substitution - Missense
GMPS	ENST00000496455	Yes	p.V613L	c.1837G>T	Unknown	Heterozygous	Unverified	Substitution - Missense
GMPS	ENST00000496455	Yes	p.P612P	c.1836G>T	Previously Reported	Heterozygous	Unverified	Substitution - coding silent
GPC3	ENST00000370818	Yes	p.V289L	c.865G>T	Previously Reported	Homozygous	Unverified	Substitution - Missense
KAT6B	ENST00000287239	Yes	p.G1134C	c.3400G>T	Previously Reported	Homozygous	Unverified	Substitution - Missense
KMT2D	ENST00000301067	Yes	p.G3195V	c.9584G>T	Unknown	Heterozygous	Unverified	Substitution - Missense
KRAS	ENST00000311936	Yes	p.G12C	c.34G>T	Confirmed Somatic	Heterozygous	Verified	Substitution - Missense
NBN	ENST00000265433	Yes	p.V153I	c.457G>A	Unknown	Heterozygous	Unverified	Substitution - Missense
NIN	ENST00000382041	Yes	p.P1987S	c.5959C>T	Unknown	Heterozygous	Unverified	Substitution - Missense
NTRK3	ENST00000394480	Yes	p.?	c.2293-10A>T	Unknown	Heterozygous	Unverified	Unknown
PDE4DIP	ENST00000369354	Yes	p.V1261L	c.3781G>T	Unknown	Heterozygous	Unverified	Substitution - Missense
PDGFRA	ENST00000257290	Yes	p.P250H	c.749C>A	Previously Reported	Heterozygous	Unverified	Substitution - Missense
PMS2	ENST00000265849	Yes	p.E491K	c.1471G>A	Unknown	Heterozygous	Unverified	Substitution - Missense
PTPRB	NM_002837.2	Yes	p.T1280M	c.3839C>T	Unknown	Heterozygous	Unverified	Substitution - Missense
RANBP2	ENST00000283195	Yes	p.D387N	c.1159G>A	Unknown	Heterozygous	Unverified	Substitution - Missense
SETBP1	ENST00000282030	Yes	p.Q1244R	c.3731A>G	Unknown	Homozygous	Unverified	Substitution - Missense
SMARCA4	ENST00000429416	Yes	p.K1566_E1567>N*	c.4698_4699GG>TT	Unknown	Homozygous	Verified	Complex - compound substitution
STK11	ENST00000326873	Yes	p.W332*	c.996G>A	Confirmed Somatic	Homozygous	Verified	Substitution - Nonsense
TP53	ENST00000269305	Yes	p.M246I	c.738G>C	Confirmed Somatic	Homozygous	Verified	Substitution - Missense
TRRAP	ENST00000355540	Yes	p.Y2267Y	c.6801C>T	Unknown	Heterozygous	Unverified	Substitution - coding silent
USP6	NM_004505.1	Yes	p.T693T	c.2079A>G	Unknown	Heterozygous	Unverified	Substitution - coding silent

Showing 1 to 39 of 39 entries

First Previous 1 Next Last

Supplementary File 1

Cell lines » Sample » Overview » MDA-MB-231

View in GRCh37 Archive

Circos | Genome Browser | Overview | Mutations | Fusions | Mutation Spectrum | Sequence Context | Heatmap | Breakpoints
Non-Coding mutation | CNV & Expr | Methylation

Show 25 entries   Search: Export: CSV TSV

Gene	Transcript	Census Gene	AA Mutation	CDS Mutation	Somatic status	Zygosity	Validated	Type
ATM	ENST00000278616	Yes	p.?	c.4237-9A>G	Unknown	Heterozygous	Unverified	Unknown
ATM	ENST00000278616	Yes	p.N1005I	c.3014A>T	Unknown	Heterozygous	Unverified	Substitution - Missense
AXIN2	ENST00000307078	Yes	p.?	c.1713-8C>T	Unknown	Heterozygous	Unverified	Unknown
BRAF	ENST00000288602	Yes	p.G464V	c.1391G>T	Confirmed Somatic	Heterozygous	Verified	Substitution - Missense
CASP8	ENST00000264275	Yes	p.L132L	c.396A>G	Unknown	Heterozygous	Unverified	Substitution - coding silent
CD79A	ENST00000221972	Yes	p.C106Y	c.317G>A	Previously Reported	Heterozygous	Unverified	Substitution - Missense
CLTCL1	ENST00000263200	Yes	p.D1485N	c.4453G>A	Previously Reported	Heterozygous	Unverified	Substitution - Missense
CRTC3	ENST00000268184	Yes	p.P578A	c.1732C>G	Previously Reported	Heterozygous	Unverified	Substitution - Missense
EXT2	ENST00000533608	Yes	p.E672E	c.2016G>A	Unknown	Heterozygous	Unverified	Substitution - coding silent
GAS7	ENST00000432992	Yes	p.?	c.1218+8G>T	Unknown	Homozygous	Unverified	Unknown
GNAS	ENST00000371085	Yes	p.N6N	c.18C>T	Unknown	Heterozygous	Unverified	Substitution - coding silent
HOXD13	ENST00000249505	Yes	p.P260P	c.780G>A	Unknown	Heterozygous	Unverified	Substitution - coding silent
KRAS	ENST00000311936	Yes	p.G13D	c.38G>A	Confirmed Somatic	Heterozygous	Verified	Substitution - Missense
NF1	ENST00000358273	Yes	p.T467fs*3	c.1398_1399insC	Unknown	Heterozygous	Unverified	Insertion - Frameshift
NF2	ENST00000338641	Yes	p.E231*	c.691G>T	Confirmed Somatic	Homozygous	Verified	Substitution - Nonsense
PBRM1	ENST00000337303	Yes	p.I228V	c.682A>G	Previously Reported	Heterozygous	Unverified	Substitution - Missense
PCSK7	ENST00000320934	Yes	p.P52R	c.155C>G	Previously Reported	Heterozygous	Unverified	Substitution - Missense
PDGFRA	ENST00000257290	Yes	p.Y172F	c.515A>T	Previously Reported	Heterozygous	Unverified	Substitution - Missense
PER1	ENST00000317276	Yes	p.E189D	c.567G>T	Unknown	Homozygous	Unverified	Substitution - Missense
SLC45A3	ENST00000367145	Yes	p.R251W	c.751C>T	Unknown	Heterozygous	Unverified	Substitution - Missense
TCEA1	ENST00000360389	Yes	p.?	c.126+7A>T	Unknown	Heterozygous	Unverified	Unknown
TP53	ENST00000269305	Yes	p.R280K	c.839G>A	Confirmed Somatic	Heterozygous	Verified	Substitution - Missense

Showing 1 to 22 of 22 entries   First Previous 1 Next Last

Supplementary File 1

Wgs » Sample » Overview » MDA-MB-435

View in GRCh37

Circos | Genome Browser | Overview | **Mutations** | Fusions | Mutation Spectrum | Non-Mutant Genes | Breakpoints | Non-Coding mutation | CNV & Expr | Methylation | Studies | References

Show 10 entries Search: Export: CSV TS

Gene	Transcript	Census Gene	AA Mutation	CDS Mutation	Somatic status	Zygosity	Validated	Type
ALK	ENST00000389048	Yes	p.R69Q	c.206G>A	Confirmed Somatic	Heterozygous	Unverified	Substitution - Missense
ARID1A	ENST00000324856	Yes	p.S1707G	c.5119A>G	Confirmed Somatic	Heterozygous	Unverified	Substitution - Missense
ATR	NM_001184	Yes	p.S601F	c.1802C>T	Confirmed Somatic	Heterozygous	Unverified	Substitution - Missense
BRAF	ENST00000288602	Yes	p.V600E	c.1799T>A	Confirmed Somatic	Heterozygous	Unverified	Substitution - Missense
BRD3	ENST00000303407	Yes	p.S609F	c.1826C>T	Confirmed Somatic	Heterozygous	Unverified	Substitution - Missense
CAMTA1	ENST00000303635	Yes	p.E205K	c.613G>A	Confirmed Somatic	Heterozygous	Unverified	Substitution - Missense
CDH1	ENST00000261769	Yes	p.Y190H	c.568T>C	Confirmed Somatic	Heterozygous	Unverified	Substitution - Missense
CDKN2A	ENST00000304494	Yes	p.?	c.150+2T>C	Confirmed Somatic	Heterozygous	Unverified	Unknown
COL1A1	ENST00000225964	Yes	p.R312C	c.934C>T	Confirmed Somatic	Homozygous	Unverified	Substitution - Missense
COL2A1	ENST00000337299	Yes	p.R989C	c.2965C>T	Confirmed Somatic	Heterozygous	Unverified	Substitution - Missense
DICER1	ENST00000343455	Yes	p.M1825I	c.5475G>A	Confirmed Somatic	Heterozygous	Unverified	Substitution - Missense
EBF1	ENST00000313708	Yes	p.G107R	c.319G>A	Confirmed Somatic	Heterozygous	Unverified	Substitution - Missense
ERC1	ENST00000397203	Yes	p.S392F	c.1175C>T	Confirmed Somatic	Heterozygous	Unverified	Substitution - Missense
KMT2C	ENST00000262189	Yes	p.E4319K	c.12955G>A	Confirmed Somatic	Heterozygous	Unverified	Substitution - Missense
PTPRK	ENST00000368226	Yes	p.H1045Y	c.3133C>T	Confirmed Somatic	Heterozygous	Unverified	Substitution - Missense
RHOH	ENST00000505618	Yes	p.R157W	c.469C>T	Confirmed Somatic	Homozygous	Unverified	Substitution - Missense
SEPT6	ENST00000343984	Yes	p.P181S	c.541C>T	Confirmed Somatic	Heterozygous	Unverified	Substitution - Missense
SPEN	ENST00000375759	Yes	p.P3345S	c.10033C>T	Confirmed Somatic	Heterozygous	Unverified	Substitution - Missense
SPEN	ENST00000375759	Yes	p.R423C	c.1267C>T	Confirmed Somatic	Heterozygous	Unverified	Substitution - Missense
TP53	ENST00000269305	Yes	p.G266E	c.797G>A	Confirmed Somatic	Heterozygous	Unverified	Substitution - Missense

Showing 1 to 20 of 20 entries

First Previous 1 Next Las

Supplementary File 1
